# Temporal trend and spatial analysis of oral cancer cases in Brazil: Correlation between socioeconomic factors and delay in diagnosis and treatment

**DOI:** 10.1111/tmi.14141

**Published:** 2025-06-22

**Authors:** Deane Cristina da Rocha Rodrigues de Oliveira, Wandklebson Silva da Paz, Márcio Bezerra‐Santos, Priscila Lima dos Santos, Débora dos Santos Tavares

**Affiliations:** ^1^ Program in Applied Health Sciences Federal University of Sergipe Lagarto Sergipe Brazil; ^2^ Program in Tropical Medicine Federal University of Pernambuco Recife Pernambuco Brazil; ^3^ Medical Science Center Federal University of Alagoas Arapiraca Alagoas Brazil; ^4^ Department of Health Education Federal University of Sergipe Lagarto Sergipe Brazil

**Keywords:** delay in diagnosis, delay in treatment, mouth neoplasms, socioeconomic factors, spatial analysis, time series studies

## Abstract

**Objective:**

To evaluate the temporal trend and spatial distribution of oral cancer cases in Brazil, and to evaluate the relationship between oral cancer cases and socioeconomic conditions.

**Methods:**

Data on oral cancer cases, between 2013 and 2019, were extracted from the Painel‐Oncologia website, and the socioeconomic indicators selected were Municipal Human Development Index and Social Vulnerability Index. The endpoints were late diagnosis (staging III and IV) and delay in treatment (>60 days), along with oral cancer prevalence. Spearman's correlation was done between oral cancer cases and Municipal Human Development Index/ Social Vulnerability Index. Temporal trends were evaluated using a segmented linear regression model. As for spatial analysis, global and local Moran indices were applied, together with spatiotemporal scan statistics, to detect risk clusters.

**Results:**

In the period studied, there was a prevalence of 5.3 oral cancer cases/100,000 inhabitants. A significant inverse correlation was found with the Social Vulnerability Index, and a direct correlation linking the Municipal Human Development Index and oral cancer cases, delayed treatment, and diagnosis cases too. An increasing trend of oral cancer prevalence rate and a stable trend of delayed diagnosis and treatment cases were observed in the country. There was a concentration of oral cancer cases in the South and Southeast regions. A high‐risk oral cancer cluster was identified covering the South and Southeast regions, and part of the Midwest region and four secondary clusters of delayed treatment cases in the Northeast region.

**Conclusion:**

There was no short‐term improvement in data related to oral cancer in Brazil, since the prevalence trend was increasing and there was a correlation with socioeconomic conditions.

## INTRODUCTION

Oral cancer (OC) is a malignant tumour that affects the lips and structures of the mouth, such as gums, buccal mucosa, palate, tongue and the floor of the mouth. Its aetiology is related to numerous risk factors divided into external (habits and living conditions, social environment, access to health services) and internal factors (genetics) [1].

Brazil has the highest incidence rate of OC in South America, comprising 3.6 cases per 100,000 inhabitants, and the second highest mortality rate, 1.5 deaths per 100,000 inhabitants [2]. National data indicate that OC is the fifth most common type of cancer in men and the tenth in women [3]. A global estimate indicates that the increase of OC incidence rates in low and medium Human Development Index (HDI) countries is likely to be accompanied by increases in mortality rates too, as observed for breast and colorectal cancer [4].

In 2012, the Brazilian government approved Law n. 12,732/12, which ensures patients initiate cancer treatment within 60 days from the diagnosis [5]. The tumours diagnosed at stages III and IV indicate that cancer is in an advanced stage and imply a late diagnosis [6]. The initiation of oncological treatment in advanced stages is a reality among OC cases in Brazil. Data from the Hospital Cancer Registry showed that more than 60% of cases of OC treated in the country between 2004 and 2015 were diagnosed in an advanced stage [7]. Delay in cancer diagnosis and treatment are often associated with unfavourable outcomes, such as lower survival rates and poor quality of life [8].

The vast territorial extension, cultural differences, and socioeconomic inequalities reinforce the detection of different health conditions, including OC risk factors, and healthcare access among the geographic regions of Brazil [9]. In addition, social inequalities were already associated with the increase in mortality from OC in Latin American countries [10], and the higher prevalence of OC cases in advanced stages in Brazil [11]. All in all, the present study sought to assess the temporal trend and spatial distribution of OC cases in Brazil, from 2013 to 2019, and to correlate them with socioeconomic indicators of the country's municipalities.

## MATERIALS AND METHODS

### Study area

Brazil is the fifth largest country by land area and is politically and administratively divided into 27 Federative Units (UF), grouped into five regions (North, Northeast, Southeast, South and Midwest) and 5570 municipalities. The country has about 207 million inhabitants [12].

### Data sources and variables

Data referring to cases of OC were obtained from PAINEL‐Oncologia [13]. These data are in the public domain and can be obtained from the website of the Information Department of Brazil's Unified National Health System (DATASUS). Categories C00 to C06 (C00 lip, C01 base of tongue, C02 other non‐specific parts of the tongue, C03 gingiva, C04 floor of the mouth, C05 palate, C06 other non‐specific parts of the mouth) of the International Statistical Classification of Diseases and Related Health Problems, 10th Revision, were selected as cause for OC.

The study variables related to OC were at obtained PAINEL‐Oncologia website. Late‐diagnosis cases were assessed by the selection of the “Stage” category, options stages III and IV. Tumours in stages III and IV indicate that the disease is in an advanced stage, with large dimensions and lymph node involvement [13]. Late‐treatment cases were selected by ticking the option “Treatment more than 60 days” in the “Treatment” category, considering the statements of Law n° 12,732/2012 [5]. Demographic data from 2013 to 2019 were obtained from the Brazilian Institute of Geography and Statistics (IBGE) website, based on data from the 2010 national population census and official intercensal estimates [14].

Thus, the study variables related to oral cancer were: (a) Prevalence rate: total number of OC cases for each year and location divided by the population for each year and location, multiplying the result by 100,000 inhabitants; (b) Late‐treatment prevalence rate: total number of OC cases treated for each year and location divided by the population for each year and location, multiplying the result by 100,000 inhabitants; (c) Late‐diagnosis prevalence rate: total number of cases of late diagnosis of OC [15] for each year and location, divided by the population for each year and location, multiplying the result by 100,000 inhabitants; (d) Mean prevalence rate: total number of OC cases for each year and location divided by the core population for the entire study period. Then, the quotient was divided by the number of years selected for each location, and the result was multiplied by 100,000 inhabitants. The average prevalence rate was also calculated for the previous 3 prevalence rates; (e) Percentage of late‐treatment OC cases: total number of late‐treatment OC cases for each year and location divided by the number of OC cases for each year and location, and multiplying the result by 100; (f) Percentage of OC cases with late diagnosis: the total number of OC cases with late diagnosis for each year and location was divided by the number of OC cases for each year and location, and the result was multiplied by 100.

Socioeconomic variables were extracted from the website of the Institute of Applied Economic Research (IPEA), also based on the 2010 Brazilian demographic census [16]. The following socioeconomic indicators referring to Brazilian municipalities were used as variables: (a) Municipal Human Development Index (MHDI), which comprises 3 sub‐indices, Education, Longevity and Income; (b) Social Vulnerability Index (SVI) which also comprises 3 sub‐indices, Urban Infrastructure, Human Capital and Income and Employment.

### Data processing and analysis

Data were stored and organised in Microsoft Office Excel 2016 software. The prevalence rate was calculated by UF of residence, geographic region of residence, and municipality of diagnosis, considering the total number of cases of OC between 2013 and 2019.

D'Agostino‐Pearson normality test was applied to assess the parametric distribution of data. Considering that the results were non‐parametric, the Spearman correlation test (Rho) was performed to verify the correlation between socioeconomic variables and the OC prevalence rate, late‐treatment prevalence rate, and late‐diagnosed prevalence rate.

Temporal trends were analysed by segmented linear regression (Joinpoint 4.9.0.0 software), UF and geographic region of residence due to the large number of Brazilian municipalities with cases of OC. The temporal trends of the OC prevalence rate (per 100,000 inhab.) and the percentage of late‐treated OC cases and late‐diagnosed OC cases were investigated. The Annual Percentage Changes (APC) was calculated for each segment and the Average Annual Percentage Changes (AAPC) for the entire period when there was more than one significant inflexion point during the studied period. APCs and AAPCs were significant when the *p*‐value <0.05. Temporal trends were classified as decreasing (APC− and *p* < 0.05), increasing (APC+ and p < 0.05) or stable (APC+ or – and *p* > 0.05).

Maps of Brazil representing OC prevalence rates in Brazilian municipalities were constructed to observe spatial distribution, along with maps representing the spatial distribution of smoothed prevalence rates, through the Local Empirical Bayesian Estimator. The indicator extracts were categorised into 6 equal ranges: 0; 0.01 to 4000/100,000 inhab.; 4000 to 8000/100,000 inhab.; 8000 to 12,000/100,000 inhab.; 12,000 to 16,000/100,000 inhab. and ≥16,000/100,000 inhab.

As for spatial analysis, the Global Moran Index of the mean prevalence rate was calculated, and then the local autocorrelation was evaluated by the Local Univariate Moran Index (Local Indicators of Spatial Association – LISA). Statistical analyses were performed using TerraView software (version 4.2.2) with a significance level of *p*‐value <0.05, and maps were prepared using QGIS software (version 3.16).

Space–time risk clusters were assessed by the space–time scan statistic (SatScan) program, using the Kulldorf method of retrospective analysis and applying the Poisson probability distribution model. The following parameters were used: aggregation time of 1 year, geographic, temporal or overlapping absence of clusters, circular clusters, maximum spatial cluster size of 50% of the population at risk and maximum temporal cluster size of 50% of the period studied. The most likely cluster (primary cluster) and secondary clusters were detected using the likelihood ratio test (LRT) and represented in the form of maps and tables. Additionally, the relative risks (RR) of the OC prevalence rate were calculated for each cluster, comparing observed and expected cases. The results were considered statistically significant when a value of *p* < 0.05 was obtained. Analyses were performed using SatScan software (version 9.6.1), and maps were elaborated using QGIS software (version 3.16).

## RESULTS

**FIGURE 1:**
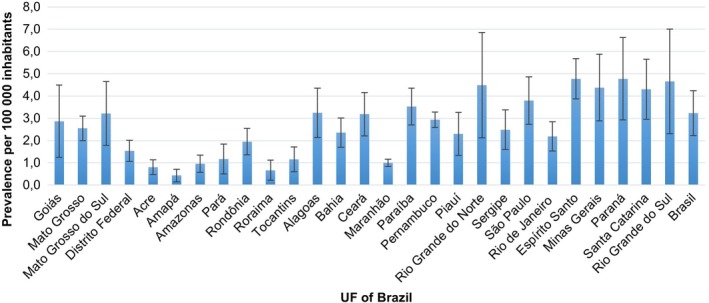
Prevalence of oral cancer by UF and Brazil, Brazil, 2013–2019.

**FIGURE 2:**
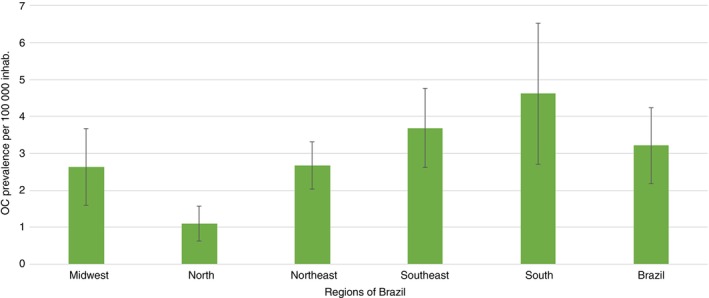
Prevalence of oral cancer by region and Brazil, Brazil, 2013–2019.

The OC prevalence rate in Brazil was 5.3 cases per 100,000 inhab., between 2013 and 2019. The UF that presented the highest and lowest prevalence rates were Espírito Santo (4.8) and Paraná (4.8) and Amapá (0.4), respectively (Figure 1). Regarding the Brazilian regions, the South region had the highest prevalence rate (4.6) and the North (1.1) the lowest one (Figure 2).

Between 2013 and 2019, 46,735 cases of OC were registered in Brazil. The highest frequencies were observed in the Southeast region (*n* = 22,254), mainly in São Paulo (*n* = 11,932) and among men (*n* = 35,645). It was found that over 27,240 cases of OC were late diagnosed, with the Southeast region (*n* = 12,683) and UF São Paulo (*n* = 6149) being the places with the highest frequencies of late diagnosis. Besides, 40,485 OC cases were late treated, with the Southeast region (*n* = 11,732) and UF São Paulo (*n* = 6297) being the places that presented the highest frequencies (Table 1).

**TABLE 1:** Frequency of the number of cases of oral cancer (Categories C0 to C06 – CID 10), according to region, FU, sex and late diagnosis, Brazil, 2013–2019.

FU	Male	Women	Total cases	Cases diagnosed late	Cases treated late	Total cases*
	*N* (%)	*N* (%)	*N* (%)	*N* (%)	*N* (%)	*N* (%)
Midwest						
Distrito Federal	237 (75)	59 (25)	316 (11)	213 (12)	191 (13)	296 (12)
Goiás	987 (72)	377 (28)	1364 (47)	754 (43)	740 (50)	1094 (45)
Mato Grosso	469 (78)	128 (22)	597 (21)	411 (24)	263 (18)	546 (22)
Mato Grosso Do Sul	484 (80)	124 (20)	608 (21)	369 (21)	277 (19)	496 (20)
Total	2177 (76)	688 (24)	2865 (100)	1747 (100)	1471 (100)	2431 (100)
North						
Acre	35 (76)	11 (24)	46 (3)	32 (4)	31 (4)	43 (4)
Amapá	15 (62,5)	9 (37,5)	24 (2)	15 (2)	19 (2)	22 (2)
Amazonas	202 (75)	67 (25)	268 (19)	84 (10)	153 (19)	244 (20)
Pará	469 (69)	214 (31)	683 (48)	398 (48)	403 (49)	522 (45)
Rondônia	185 (76)	57 (24)	242 (17)	176 (21)	116 (14)	224 (18)
Roraima	24 (96)	1 (4)	25 (2)	17 (2)	20 (2)	24 (2)
Tocantins	88 (70)	37 (30)	125 (9)	101 (12)	74 (9)	112 (9)
Total	1017 (72)	396 (28)	1413 (100)	823 (100)	816 (100)	1221 (100)
Northeast						
Alagoas	469 (62)	289 (38)	758 (7)	444 (7)	415 (7)	633 (7)
Bahia	1849 (74)	634 (26)	2483 (23)	1924 (28)	1607 (27)	2281 (24)
Ceará	1456 (73)	548 (27)	2004 (19)	1256 (19)	956 (16)	1775 (19)
Maranhão	350 (72)	137 (28)	487 (5)	295 (4)	263 (4)	455 (5)
Paraíba	697 (71)	324 (29)	984 (9)	612 (9)	712 (12)	906 (10)
Pernambuco	1430 (74)	500 (26)	1930 (5)	1185 (17)	1255 (21)	1827 (19)
Piauí	360 (69)	160 (31)	520 (18)	343 (5)	208 (3)	444 (5)
Rio grande do Norte	713 (65)	375 (35)	1088 (10)	516 (8)	382 (6)	935 (10)
Sergipe	280 (71)	114 (29)	394 (4)	213 (3)	201 (3)	254 (3)
Total	7604 (71)	3111 (29)	10,648 (100)	6788 (100)	5999 (100)	9510 (100)
Southeast						
Espírito Santo	1035 (78)	287 (22)	1322 (6)	1012 (8)	788 (7)	1220 (6)
Minas Gerais	4900 (76)	1530 (24)	6430 (29)	3926 (31)	3088 (26)	5501 (29)
Rio de Janeiro	1874 (73)	694 (27)	2570 (12)	1596 (13)	1559 (13)	2274 (12)
São Paulo	9463 (79)	2478 (21)	11,932 (54)	6149 (48)	6297 (54)	10,205 (53)
Total	17,272 (78)	4989 (22)	22,254 (100)	12,683 (100)	11,732 (100)	19,200 (100)
South						
Paraná	2983 (79)	782 (21)	3765 (39)	2169 (42)	1324 (34)	3276 (40)
Rio Grande do Sul	2898 (79)	760 (21)	3658 (39)	1689 (32)	1563 (4)	3031 (37)
Santa Catarina	1693 (81)	399 (19)	2092 (22)	1342 (26)	1060 (27)	1816 (22)
Total	7574 (79)	1941 (21)	9535 (100)	5200 (100)	3947 (100)	8123 (100)
Brazil	35,645 (76)	11,125 (24)	46,735 (100)	27,241	23,965	40,485

*Source*: Oncology Panel: DATASUS.

* In this total of cases, cases without information were excluded.

Spearman's correlation test indicated a correlation between socioeconomic variables and OC prevalence rate, late‐treatment prevalence rate and late‐diagnosed prevalence rate: there was an inversely and directly significant correlation (*p*‐value <0.0001) between SVI and MHDI, respectively (Table 2).

**TABLE 2:** Correlation between population socioeconomic variables and the prevalence of oral cancer, Brazil, 2013–2019.

Social determinants of health (SDH)	Rho	CI 95%	*p*‐value*
Total cases			
Social Vulnerability Index (SVI)	−0.3538	−0.3772; −0.3299	<0.0001
SVI – Urban infrastructure	−0.2648	−0.2897; −0.2394	<0.0001
SVI – Human capital	−0.3469	−0.3704; −0.3229	<0.0001
SVI – Income and Work	−0.3239	−0.3479; −0.2995	<0.0001
Municipal Human Development Index (MHDI)	0.3244	0.3000; 0.3484	<0.0001
MHDI – Longevity	0.2999	0.2751; 0.3243	<0.0001
MHDI – Education	0.2933	0.2684; 0.3178	<0.0001
MHDI – Income	0.3168	0.2923; 0.3409	<0.0001
Lately treated cases			
Social Vulnerability Index (SVI)	−0.2298	−0.2552; −0.2040	<0.0001
SVI – Urban infrastructure	−0.1687	−0.1949; −0.1423	<0.0001
SVI – Human capital	−0.2279	−0.2533; −0.2021	<0.0001
SVI – Income and Work	−0.208	−0.2337; −0.1820	<0.0001
Municipal Human Development Index (MHDI)	0.2222	0.1964; 0.2478	<0.0001
MHDI – Longevity	0.1979	0.1718; 0.2238	<0.0001
MHDI – Education	0.2038	0.1777; 0.2295	<0.0001
MHDI – Income	0.2155	0.1896; 0.2411	<0.0001
Late‐diagnosed cases			
Social Vulnerability Index (SVI)	−0.2622	−0.2872; −0.2368	<0.0001
SVI – Urban infrastructure	−0.1959	−0.2218; −0.1698	<0.0001
SVI – Human capital	−0.2614	−0.2864; −0.2360	<0.0001
SVI – Income and Work	−0.2338	−0.2592; −0.2080	<0.0001
Municipal Human Development Index (MHDI)	0.2463	0.2207; 0.2715	<0.0001
MHDI – Longevity	0.2265	0.2007; 0.2520	<0.0001
MHDI – Education	0.2221	0.1962; 0.2476	<0.0001
MHDI – Income	0.2396	0.2140; 0.2649	<0.0001

Abbreviations: CI, confidence interval; Rho: Spearman correlation test.

* Statistically significant (*p* < 0.05).

The temporal trend of OC prevalence rate was increasing in 16 UF and stable in 11: Goiás (AAPC = 22.1%) and Pará (AAPC = 21%) presented the highest annual increases (*p* < 0.05) during the period studied (2013–2019). No UF showed a decreasing trend. There was a stable trend in the percentage of late‐diagnosed OC cases in 19 UF, an increasing trend in 2 and a decreasing trend in 6 UF: Roraima (APC = −9.75%) presented the highest annual variations (*p*‐value <0.05) (Table 3). At last, there was a stable trend in the percentage of late‐treated OC cases in 23 UF, increasing in 1 UF and decreasing in 4. The UF Bahia (APC = −3.2%) had the highest annual changes (*p*‐value <0.05) (Table 4).

**TABLE 3:** Time trend of oral cancer prevalence rate in Brazil by FU and region from 2013 to 2019.

Region/Federal Unit (FU)	Period	Prevalence rate	Segmented period APC** (CI*** 95%)	Trend	Total period AAPC**** (CI** 95%)	Trend
Midwest	2013–2017	2.4–2.3	−1.2 (−20.2 to 22.4)	Stable	13.9* (4.0–24.7)	Increscent
	2017–2019	2.3–4.0.8	51.3 (−0.2 to 129.3)			
Distrito Federal	2013–2019	1.9–2	2.7 (−8.0 to 14.8)	Stable	–	–
Goiás	2013–2017	1.88–2.5	5.0 (−12.3 to 25.4)	Stable	**22.1** * (13.7–31.2)	**Increscent**
	2017–2019	2.5–6.4	**65.4** * (21.4–125.3)	**Increscent**		
Mato Grosso	2013–2019	3–2.9	3.2 (−6.2 to 13.5)	Stable	–	–
Mato Grosso do Sul	2013–2016	3.3–1.8	−19.1 (−49.0 to 28.2)	Stable	10.1 (−3.8 to 25.9)	Stable
	2016–2019	1.8–5.9	**49.8** * (3.6–116.7)	**Increscent**		
Northeast	2013–2017	2.2–2.2	1.7* (1.5–2.0)	Increscent	9.7* (9.6–9.8)	Increscent
	2017–2019	2.2–3.9	27.5* (26.9–28.3)	Increscent		
Alagoas	2013–2019	3–5.6	11.7 (−0.9 to 25.9)	Stable	–	–
Bahia	2013–2019	1.7–3.6	**13.4** * (8.0–19.0)	**Increscent**	–	–
Ceará	2013–2019	2.4–4.6	**12.9** * (4.6–21.9)	**Increscent**	–	–
Maranhão	2013–2019	0.9–1.2	4.3 (−2.4 to 11.4)	Stable	–	–
Paraíba	2013–2019	2.7–4.6	**9.8** * (2.8–17.4)	**Increscent**	–	–
Pernambuco	2013–2019	2.9–3.3	0.4 (−5.6 to 6.8)	Stable	–	–
Piauí	2013–2017	2.4–1.3	−9.8 (−27.3 to 12.0)	Stable	**10.9** * (1.4–21.4)	**Increscent**
	2017–2019	1.3–4.2	**67.7** * (11.5–152.0)	**Increscent**		
Rio Grande do Norte	2013–2017	3.6–2.6	1.1 (−11.0 to 15.0)	Stable	**18.7** * (13.1–24.5)	**Increscent**
	2017–2019	2.6–9.0	**63.3** * (35.9–96.1)	**Increscent**		
Sergipe	2013–2019	1.7–2.6	−0.4 (−16.6 to 18.9)	Stable	–	–
North	2013–2019	0.9–2.1	19.7* (7.5–33.2)	Increscent	–	–
Acre	2013–2019	1.3–0.8	−7.8 (−19.5 to 5.6)	Stable	–	–
Amapá	2013–2019	0.3–0.6	−2.0 (−19.1 to 18.6)	Stable	–	–
Amazonas	2013–2017	1.1–0.7	−9.9 (−44.2 to 45.4)	Stable	8.5 (−13.6 to 36.2)	Stable
	2017–2019	0.7–1.7	57.3 (−50 to 398.4)	Stable		
Pará	2013–2017	0.8–1	2.1 (−33.6 to 56.9)	Stable	**21.0** * (1.1–44.8)	**Increscent**
	2017–2019	0.8–2.6	69.9 (−24.8 to 283.8)	Stable		
Rondônia	2013–2019	1.1–2.6	**12.5** * (3.2–22.7)	**Increscent**	–	–
Roraima	2010–2017	0.2–0.7	−5.1 (−19.8 to 12.3)	Stable	–	–
Tocantins	2013–2019	0.9–2.2	**20.4** * (8.6–33.4)	**Increscent**	–	–
Southeast	2013–2019	3.2–4.8	8.6* (2,3–15,4)	Increscent	–	–
Espírito Santo	2013–2017	4.1–4.5	1.4 (−5.2 to 8.4)	Stable	**7.8** * (4.6–11.2)	**Increscent**
	2017–2019	4.1–6.6	**22.0** * (4.8–41.9)	**Increscent**		
Minas Gerais	2013–2017	3.4–3.6	3.7 (−1.6 to 9.1)	Stable	**13.9** * (11.5–16.3)	**Increscent**
	2017–2019	3.6–7.2	**37.5** * (25.5–50.7)	**Increscent**		
Rio de Janeiro	2013–2017	2–1.8	−3.1 (−17.5 to 13.8)	Stable	**10.1** * (2.1–18.7)	**Increscent**
	2017–2019	1.8–3.6	42.1 (−2.8 to 107.8)	Stable		
São Paulo	2013–2017	3.4–3.2	−0.4 (−3.7 to 3.0)	Stable	**10.3** * (8.7–11.9)	**Increscent**
	2017–2019	3.2–5.8	**35.2** * (26.4–44.6)	**Increscent**		
South	2013–2017	3.8–3.6	−0.2 (−7.8 to 8.1)	Stable	15.3* (11.6–19.0)	Increscent
	2017–2019	3.6–8.5	53.7* (34.0–76.3)	Increscent		
Paraná	2013–2017	4.1–3.7	0.1 (−16.2 to 19.6)	Stable	**14.2** * (6.1–22.8)	**Increscent**
	2017–2019	3.7–8.4	**48.4** * (7.6–104.8)	**Increscent**		
Rio Grande do Sul	2013–2017	3.7–3.4	−1.5 (−8.8 to 6.5)	Stable	**18.2** * (14.7–21.8)	**Increscent**
	2017–2019	3.4–9.6	**70.1** * (50.6–92.1)	**Increscent**		
Santa Catarina	2013–2017	3.5–3.7	1.6 (−5.7 to 9.4)	Stable	**12.8** * (8.9–15.8)	**Increscent**
	2017–2019	3.7–6.9	**37.3** * (19.9–57.2)	**Increscent**		
Brazil	2013–2017	2.7–2.7	0.9 (−4.1 to 6.1)	Stable	12.5* (10.1–14.9)	Increscent
	2017–2019	2.7–5.3	40.0*(27.1–54.3)	Increscent		

Abbreviations: AAPC, Annual Average Percentage Changes; APC, Annual Percentage Changes; CI, confidence interval.

* Statistically significant (*p*‐value <0.05, in bold).

**TABLE 4:** Time trend in the percentage of late‐treated cases in Brazil by FU and region, from 2013 to 2019.

Region/Federal Unit (FU)	Period	Percentage of late treatment	Segmented period APC (CI 95%)	Trend	Total period AAPC**** (CI*** 95%)	Trend
Midwest	2013–2019	58–53	−0.4 (−6.5 to 6.0)	Stable	–	–
Distrito Federal	2013–2019	72–42	−2.2 (−14.0 to 11.2)	Stable	–	–
Goiás	2013–2019	63–57	−1.4 (−9.0 to 6.7)	Stable	–	–
Mato Grosso	2013–2019	50–40	−1.7 (−10.1 to 7.4)	Stable	–	–
Mato Grosso do Sul	2013–2019	52–61	3.8 (−1.1 to 9.0)	Stable	–	–
Northeast	2013–2019	63–53	−2.1 (−6.0 to 1.9)	Stable	–	–
Alagoas	2013–2019	72–56	−3.1 (−7.8 to 1.8)	Stable	–	–
Bahia	2013–2019	75–64	**−3.2** * (−4.9 to −1.4)	**Decrescent**	–	–
Ceará	2013–2019	46–49	0.5 (−4.3 to 5.7)	Stable	–	–
Maranhão	2013–2019	64–52	−3.2 (−8.2 to 2.1)	Stable	–	–
Paraíba	2013–2019	74–74	−1.4 (−9.0 to 6.9)	Stable	–	–
Pernambuco	2013–2019	71–58	−1.4 (−7.7 to 5.3)	Stable	–	–
Piauí	2013–2019	32–30	6.0 (−5.6 to 19.0)	Stable	–	–
Rio Grande do Norte	2013–2019	46–33	−4.9 (−10.9 to 1.6)	Stable	–	–
Sergipe	2013–2019	70–65	−1.0 (−10.0 to 8.9)	Stable	–	–
North	2013–2019	70–59	−1.9 (−4.2 to 0.5)	Stable	–	–
Acre	2013–2019	22–80	−4.3 (−17.3 to 10.7)	Stable	–	–
Amapá	2013–2019	50–75	−5.2 (−13.8 to 4.3)	Stable	–	–
Amazonas	2013–2019	88–53	−5.2 (−13.8 to 4.3)	Stable	–	–
Pará	2013–2019	79–66	**−2.8** * (−5.5 to −0.1)	**Decrescent**	–	–
Rondônia	2013–2019	37–62	**11.4** * (1.3–22.6)	**Increscent**	–	–
Roraima	2013–2019	100–50	−3.5 (−15.1 to 9.7)	Stable	–	–
Tocantins	2013–2019	46–38	−2.5 (−13.0 to 9.2)	Stable	–	–
Southeast	2013–2019	55–54	−0.9 (−5.4 to 3.9)	Stable	–	–
Espírito Santo	2013–2019	62–52	−0.2 (−6.7 to 6.7)	Stable	–	–
Minas Gerais	2013–2019	58–52	**−2.5** * (−4.9 to −0.0)	**Decrescent**	–	–
Rio de Janeiro	2013–2019	7–64	−2.7 (−9.2 to 4.3)	Stable	–	–
São Paulo	2013–2019	64–54	−2.0 (−5.9 to 2.0)	Stable	–	–
South	2013–2019	54–69	2.6 (−3.5 to 9.2)	Stable	–	–
Paraná	2013–2019	44–3	−0.2 (−6.5 to 6.5)	Stable	–	–
Rio Grande do Sul	2013–2019	56–40	−4.0 (−10.2 to 2.6)	Stable	–	–
Santa Catarina	2013–2019	70–49	**−5.5** * (−7.7 to −3.3)	**Decrescent**	–	–
Brazil	2013–2017	57–65	2.6 (−4.3 to 9.9)	Stable	−3.5 (−7.7 to 0.8)	Stable
	2017–2019	65–48	−14.7 (−34.1 to 10.3)	Stable		

Abbreviations: AAPC, Annual Average Percentage Changes; APC, Annual Percentage Changes; CI, confidence interval.

* Statistically significant (*p*‐value <0.05, in bold).

All Brazilian geographic regions had an increasing trend in OC prevalence rate of and at a national level, the trend was also increasing (AAPC = 12.5%) (Table 3). The North (APC = −2.8%) and Northeast (AAPC = −1.5%) regions presented a stable trend in the percentage of late‐diagnosed OC cases, and the Midwest (AAPC = −6.9%), South (AAPC = −.9%) and Southeast (AAPC = −4.8%) regions, a decreasing trend. At a national level, the percentage late‐diagnosed OC cases displayed a decreasing trend (AAPC = −4.8%) (Table 4). All Brazilian regions exhibited a stable trend in the percentage of late‐treated OC cases, and at the national level, the trend was also stable (AAPC = −3.5%) (Table 5).

**TABLE 5:** Time trend in the percentage of late‐diagnosed oral cancer cases in Brazil by FU, from 2013 to 2019.

Region/Federal Unit (FU)	Period	Late‐diagnosis percentage	Segmented period APC (CI 95%)	Trend	Total period AAPC (CI 95%)	Trend
Midwest	2013–2017	84–76	−2.7* (−1.9 to −13.9)	Decrescent	−6.9* (−7.5 to −6.3)	Decrescent
	2017–2019	76–57	−14.8* (−18.0 to 11.5)	Decrescent		
Distrito Federal	2013–2019	84–75	−2.6 (−8.4 to 3.4)	Stable	–	–
Goiás	2013–2019	83–50	**−6.6** * (−12.2 to −0.5)	**Decrescent**	–	–
Mato Grosso	2013–2019	87–53	−5.7 (−11.3 to 0.4)	Stable	–	–
Mato Grosso do Sul	2013–2019	82–65	−3.1 (−9.7 to 4.0)	Stable	–	–
Northeast	2013–2017	71–77	2.2 (−1.4 to 5.8)	Stable	−1.5 (−3.6 to 0.6)	Stable
	2017–2019	77–63	−8.4 (−18.9 to 3.4)	Stable		
Alagoas	2013–2019	80–75	−1.0 (−4.2 to 2.4)	Stable	–	–
Bahia	2013–2019	85–78	**−1.7** * (−3.2 to −0.1)	**Decrescent**	–	–
Ceará	2013–2019	69–60	−0.4 (−5.2 to 4.7)	Stable	–	–
Maranhão	2013–2019	81–51	−4.5 (−9.7 to 1)	Stable	–	–
Paraíba	2013–2019	79–57	−4.7 (−11.2 to 2.2)	Stable	–	–
Pernambuco	2013–2019	49–68	**7.0** * (2.4–11.8)	**Crescente**	–	–
Piauí	2013–2019	87–46	−3.7 (−10.8 to 3.9)	Stable	–	–
Rio Grande do Norte	2013–2019	66–41	−6.5 (−14.7 to 2.5)	Stable	–	–
Sergipe	2013–2019	64–75	4.0 (−4.3 to 13.0)	Stable	–	–
North	2013–2019	67–57	−2.8 (−8.6 to 3.4)	Stable	–	–
Acre	2013–2019	100–80	**−4.7** * (−8.7 to −0.6)	**Decrescent**	–	–
Amapá	2013–2019	500–25	2.8 (−15.5 to 25)	Stable	–	–
Amazonas	2013–2019	61–35	−11.3 (−28.6 to 10.1)	Stable	–	–
Pará	2013–2019	63–63	**−0.31** * (−6.5 to 6.7)	**Decrescent**	–	–
Rondônia	2013–2019	89–74	−3.1 (−6.4 to 0.3)	Stable	–	–
Roraima	2010–2017	100–50	**−9.75** * (−15.8 to −3.3)	**Decrescent**	–	–
Tocantins	2013–2019	54–56	−2.0 (−13.2 to 10.7)	Stable	–	–
Southeast	2013–2017	72–74	0.7 (−1.6 to 3.0)	Stable	−4.8* (−6.3 to −3.3)	Decrescent
	2017–2019	74–54	−14.9* (−22.4 to −6.7)	Decrescent		
Espírito Santo	2013–2019	88–88	**1.9** * (−3.5 to −0.2)	**Increscent**	–	–
Minas Gerais	2013–2019	79–58	**−4.2** * (−7.9 to −0.3)	**Decrescent**	–	–
Rio de Janeiro	2013–2019	82–54	−4.5 (−9.0 to 0.1)	Stable	–	–
São Paulo	2013–2019	65–49	−3.1 (−8.0 to 2.2)	Stable	–	–
South	2013–2017	74–75	−0.3 (−1.2 to 0.5)	Stable	−6.9* (−7.4 to −6.3)	Decrescent
	2017–2019	75–49	−18.7* (−21.6 to −15.6)	Decrescent		
Paraná	2013–2019	78–50	−5.0 (−10.7 to 1.0)	Stable	–	–
Rio Grande do Sul	2013–2019	64–44	−4.3 (−9.3 to 0.9)	Stable	–	–
Santa Catarina	2013–2019	84–57	−4.5 (−9.5 to 0.8)	Stable	–	–
Brazil	2013–2017	73–75	0.4 (−1.4 to 2.0)	Stable	−4.8* (−5.8 to −3.7)	Decrescent
	2017–2019	75–55	−14.2* (−19.8 to 8.3)	Decrescent		

Abbreviations: AAPC, Annual Average Percentage Changes; APC, Annual Percentage Changes; CI, confidence interval.

* Statistically significant (*p*‐value <0.05, in bold).

**FIGURE 3:**
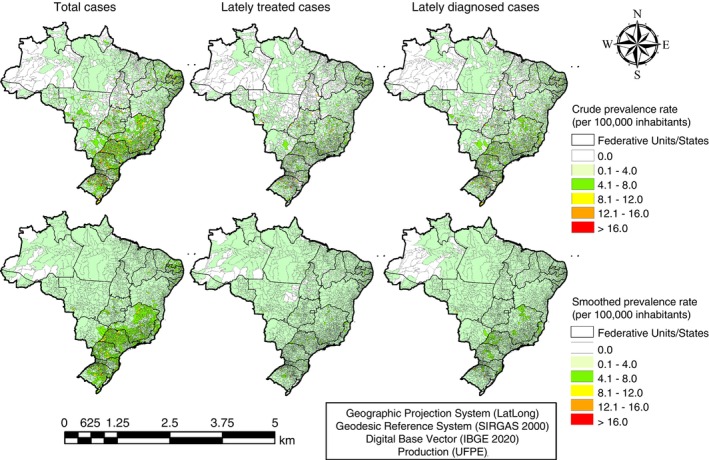
Space–time distribution of the prevalence rate of total cases, late‐treated cases, and late‐diagnosed cases of oral cancer, Brazil, 2013–2019.

Two hundred and fifty‐six Brazilian municipalities had at least one case per year of OC notified. Considering the crude and smoothed prevalence rates of total cases, a concentration of OC cases is observed in the South and Southeast regions and in some UFs located in the Northeast region. The smoothed rate revealed a concentration of late‐treatment prevalence rate OC cases in some municipalities in the South and Southeast regions. Late‐diagnosed prevalence rate of OC cases are concentrated in municipalities located in the South and Southeast regions as well (Figure 3).

**FIGURE 4:**
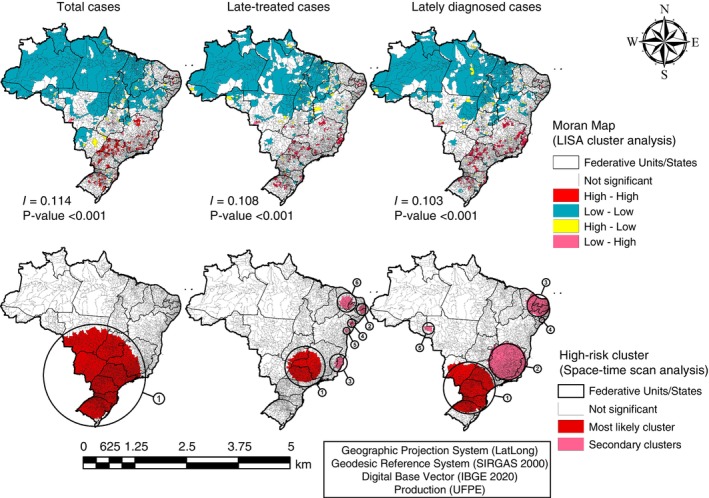
Moran Map (LISA Cluster Analysis) and High Risk Analysis (Spatial‐Temporal Scan Anaes, and late‐diagnosed cases, Brazil, 2013‐2019.

The spatial autocorrelation analysis revealed a concentration of municipalities with a high risk in the South and Southeast regions, pointing to a cluster more likely to have cases of OC in these regions. As for, there was also a cluster with a greater probability of having late‐treated OC cases among the UF São Paulo, Mato Grosso do Sul, Goiás and Minas Gerais, along with 5 secondary clusters (Table 6, Figure 4). The space–time analysis revealed a risk cluster more likely to display late‐diagnosed OC cases, located between the UF Rio Grande do Sul, Paraná, São Paulo and Mato Grosso do Sul, and also 4 secondary clusters (Table 6) (Figure 4).

**TABLE 6:** Space–time clusters of annual oral cancer prevalence rates in Brazilian municipalities between 2013 and 2019.

Clusters	Time interval	Municipalities	Observed cases	Expected cases	RR	Annual detection coefficient	LRT	*p*
Total cases								
1	2018–2019	2623	12,162	6802	2.06	5.80	2083.53	<0.0001*
Late‐treated cases								
1	2015–2017	757	1781	1161	1.58	2.60	150.13	<0.0001*
2	2014–2016	141	683	422	1.64	2.70	69.13	<0.0001*
3	2016–2018	70	344	173	2.00	3.30	65.80	<0.0001*
4	2013–2015	24	114	54	2.07	3.50	24.20	<0.0001*
5	2017–2018	30	52	23	2.19	3.70	12.46	0.211
6	2018–2019	150	143	99	1.44	2.40	8.52	0.999
Late‐diagnosed cases								
1	2013–2015	1540	3165	265	1.60	3.00	274.86	<0.0001*
2	2011–2013	2395	2410	1546	1.61	3.00	219.90	<0.0001*
3	2017–2019	585	1209	996	1.22	2.40	22.13	<0.0001*
4	2015–2017	9	54	21	2.57	5.0	17.99	<0.0001*
5	2016–2018	9	34	15	2.27	4.40	8.82	0.996

Abbreviations: LRT, likelihoodratiotest; RR, relativerisk.

* Statisticallysignificant (*p*‐value<0.05).

## DISCUSSION

To the best of our knowledge, this is the first study to use spatial and temporal trend analysis tools to assess, at a national level, cases of OC from the perspective of delay in diagnosis and treatment and considering socioeconomic indicators. Temporal trend analyses showed an increasing movement for the overall prevalence of OC and stability for the percentage of cases with late diagnosis and treatment. In addition, the spatial analysis revealed secondary risk clusters for cases with late diagnosis and treatment in the North and Northeast regions. On the other hand, a correlation was found between socioeconomic variables and the three mean prevalence rates (OC prevalence rate, late‐treatment prevalence rate, and late‐diagnosis prevalence rate). Taken together, these results revealed a difference in the behaviour of these variables among the different geographic regions displaying diverse socioeconomic indices, indicating a worrying scenario of OC in Brazil.

It was found that 27,241 thousand cases (67.29%) of oral cancer were diagnosed in advanced stages and that the trend of late‐diagnosed OC cases was stable in 19 UF, from 2013 to 2019. The difficulty in diagnosing early‐stage cancer cases is shared by other countries [8, 17, 18]. In a study carried out in Poland, with data from 2008 to 2018, it was found that of the 305 cases of OC analysed, 73.5% were already in advanced stages [8]. A study based on data from the Taiwanese cancer registry, from 2004 to 2010, found that 52% of cases were in advanced stages [18].

There was an inversely and directly significant correlation between the SVI and the MHDI, respectively, with the OC prevalence rate, late‐treatment rate and late‐diagnosed rate. These results are in accordance with the report of Harris et al. (2021), which, after investigating longitudinal trends based on the global burden of diseases caused by lip and mouth cancer, showed that the primary predictor variable was the HDI. The studied countries with high and medium HDI experienced a disproportionate increase in the disease burden of lip and oral cavity cancer [19].

Another study has also found a relationship between per capita income and the rate of deaths from OC, and suggested that cancer is linked to economic development, considering that in capitals with a high MHDI, the population is more susceptible to risk factors due to longer life expectancy and because they have a more accurate registration of health occurrences [20]. In Brazil, life expectancy in the South region, for example, is higher than in the Northeast region of Brazil [21]. Regarding the registration of health information, despite the improvement of the information system observed during recent years in Brazil, there is still underreporting in the most remote regions [21, 22].

The results of the present study indicate that the publication of a single legal provision is not enough to transform health practices [23]. The time trend of OC treated 60 days after the diagnosis was stable in 23 UF, and at the national level too, showing that the “60‐day Law” (No 12.732/2012) did not change the cancer scenario discussed herein, and yet that there is no prospect of change in the short term if measures to control and combat cancer are not encouraged. The relationship between socioeconomic conditions and OC is also attested by temporal trends and space–time analyses by geographic region, revealing an increasing trend of OC prevalence rate in the Northeast, a stable trend in late‐diagnosis rate in the North and Northeast, along with four secondary risk clusters for late‐treated cases and two for late‐diagnosed cases in the Northeast region. These two regions concentrate on municipalities with lower HDI and higher IVS.

Although the Brazilian population has universal health coverage, access to specialised care is still restricted to the most economically advantaged. The fact is that the Southeast region has greater technological support/infrastructure for cancer treatment, which is why many people from other regions migrate in search of better medical treatment. Considering that the highest demographic densities in the country are concentrated in this region, the centre of specialised care is strategically located there [24]. As the analysis of these data is recent in the literature, it was not possible to compare them with other studies of OC time trend analyses.

Among the main factors that may contribute to the delay in the diagnosis of OC are the population's lack of knowledge about the disease [24] and the low level of education [25, 26]. Several factors can influence care for patients with cancer, such as lack of information about the severity of OC, barriers in public service, errors in the initial diagnosis, depreciation of initial lesions, both by the patient and by the health professional, gaps in professional training, agility in service, availability of resources and professionals [27]. Late diagnosis and treatment cases increase the need for care, outpatient treatment costs, or prolonged hospitalisation, impacting the health system and the most economically disadvantaged [24, 28].

Although municipalities in the South and Southeast regions, with the highest MHDI and lowest IVS, had a greater OC prevalence rate, the municipalities with the lowest MHDI and highest IVS were more likely to have marked late‐treatment and late‐diagnosed prevalence rates. These rates may reflect OC mortality and higher mortality trends. These results converge with the study conducted by Pereira et al. (2023) that reported high mortality rates from OC in the South and Southeast regions of Brazil, between 2010 and 2019, and significant increasing trends in OC mortality rates in the North and Northeast regions. They also revealed an inverse relationship between the OC mortality rate temporal trend and socioeconomic indicators, such as HDI [29].

Cluster analysis and space–time scan analysis reported herein indicated the presence of a cluster more likely to present cases of OC in the South and Southeast regions. These results are similar to INCA estimates, which show that the Southeast region concentrates more than 60% of cancer incidence rates, followed by the Northeast (27.8%) and South (23.4%) regions, and that OC among men is the fifth most frequent type of cancer in the Southeast region and the sixth in the South region [30]. In addition, another study with national data reported a higher number of hospitalisations and frequency of deaths from OC in the Southeast and South regions of Brazil [12].

These findings can be attributed to living standards adopted in large cities in relation to work conditions, food availability and consumption patterns, which expose individuals to environmental carcinogens (chemical, physical and biological agents) that may be linked to the industrialisation process/socioeconomic development too, and the predominance of a modern lifestyle with harmful habits (such as smoking, excessive alcohol consumption, obesity) [27, 31, 32]. In addition, the increase in life expectancy, mostly in large urban centres, should be considered.

Finally, the present study presents limitations inherent to the methodology that must be considered. The use of the health information system database as a secondary data source should be analysed in terms of record reliability. Therefore, there is the possibility of bias regarding the quantity and quality of information; that is, cancer cases may be under‐ or over‐reported in some regions, or even a late registration in the system may be present. Notwithstanding, the data presented in the PAINEL‐Oncologia refer exclusively to users of the Unified Health System (SUS). Although approximately 85% of cancer cases diagnosed in the SUS are displayed in the PAINEL‐Oncologia [22], there were registered cases lacking information regarding staging and treatment time. However, the importance of this tool must be highlighted, as it can support SUS managers in monitoring the delay in starting treatment and in making decisions [22, 33].

The present study was restricted to the pre‐COVID‐19 pandemic period due to the effect of social isolation measures and redirection of health services to combat the infection, which probably affected cancer management, including OC [34], making the epidemiological scenario even worse in Brazil, as already reported in other diseases such as non‐communicable diseases, neglected tropical Diseases and heart disease [35, 36]. As this is an ecological study, it does not have an individualised analysis and detailing, and the phenomenon of ecological fallacy may occur if its findings are interpreted at individual levels. However, despite the limitations, this study provides relevant data on the diagnosis and treatment of OC in Brazil. The analysis of all cases of OC diagnosed in Brazil from 2003 to 2019, by different analysis tools, allowed the phenomenon to be observed from different points of view, which contributes to the unique evaluation of the OC scenario in Brazil.

## CONCLUSION

All in all, we found that living conditions which are directly associated with social determinants of health can play an important role in the development of OC, based on the significant correlation between socioeconomic variables and OC rates assessed and high‐risk clusters in specific areas of the country. Concurrently, an increasing trend of the OC prevalence rate was verified along with a decreasing and stable trend of late‐treated and late‐diagnosed OC cases, respectively. This epidemiological scenario may provide evidence for policy‐makers to prioritise actions towards the fight against this preventable, treatable, and curable cancer, in the vast majority of cases, if early diagnosed.

## CONFLICT OF INTEREST STATEMENT

The authors declare no conflict of interest. The funders had no role in the design of the study; in the collection, analyses, or interpretation of data; in the writing of the manuscript; or in the decision to publish the results.
